# Advanced Glycation End Products and Oxidative Stress in Type 2 Diabetes Mellitus

**DOI:** 10.3390/biom5010194

**Published:** 2015-03-16

**Authors:** Kerstin Nowotny, Tobias Jung, Annika Höhn, Daniela Weber, Tilman Grune

**Affiliations:** Department of Molecular Toxicology, German Institute of Human Nutrition Potsdam-Rehbruecke, Arthur-Scheunert-Allee 114-116, 14558 Nuthetal, Germany; E-Mails: Kerstin.Nowotny@dife.de (K.N.); Tobias.Jung@dife.de (T.J.); Annika.Hoehn@dife.de (A.H.); Daniela.Weber@dife.de (D.W.)

**Keywords:** advanced glycation end products, oxidative stress, type 2 diabetes mellitus, insulin resistance, β cell dysfunction, diabetic complications

## Abstract

Type 2 diabetes mellitus (T2DM) is a very complex and multifactorial metabolic disease characterized by insulin resistance and β cell failure leading to elevated blood glucose levels. Hyperglycemia is suggested to be the main cause of diabetic complications, which not only decrease life quality and expectancy, but are also becoming a problem regarding the financial burden for health care systems. Therefore, and to counteract the continually increasing prevalence of diabetes, understanding the pathogenesis, the main risk factors, and the underlying molecular mechanisms may establish a basis for prevention and therapy. In this regard, research was performed revealing further evidence that oxidative stress has an important role in hyperglycemia-induced tissue injury as well as in early events relevant for the development of T2DM. The formation of advanced glycation end products (AGEs), a group of modified proteins and/or lipids with damaging potential, is one contributing factor. On the one hand it has been reported that AGEs increase reactive oxygen species formation and impair antioxidant systems, on the other hand the formation of some AGEs is induced *per se* under oxidative conditions. Thus, AGEs contribute at least partly to chronic stress conditions in diabetes. As AGEs are not only formed endogenously, but also derive from exogenous sources, *i.e.*, food, they have been assumed as risk factors for T2DM. However, the role of AGEs in the pathogenesis of T2DM and diabetic complications—if they are causal or simply an effect—is only partly understood. This review will highlight the involvement of AGEs in the development and progression of T2DM and their role in diabetic complications.

## 1. Introduction

With around 350 million cases in 2014 [1], type 2 diabetes mellitus (T2DM) is one of the most frequent diseases throughout the world. This number is predicted to increase dramatically in the coming years, resulting in serious health and economic challenges. In general, diabetes mellitus is a group of metabolic diseases in which the pancreas is not able to produce insulin, insulin production is not sufficient or cells cannot effectively use this hormone [2]. In T2DM, the body is *per se* able to produce insulin. However, several processes induce abnormalities in a manner that either hormone production is insufficient or cells are unable to mediate the effects of insulin. As insulin is required for an efficient cellular uptake of glucose to convert it into energy, the ineffectiveness of insulin causes elevated blood glucose levels (hyperglycemia). Hyperglycemia is estimated to be one major factor contributing to diabetic complications including diseases which affect the cardiovascular and nervous system, eyes or kidneys. Every year, 4.9 million people die from diabetes [1] some 50% of them by cardiovascular complications [3]. To prevent diabetic complications, an early diagnosis is very important. However, the high prevalence of T2DM, its morbidity as well as mortality rates are at least partly due to the fact, that most people only recognize this disease in a state when symptoms already occur. This indicates that the diagnostic and therefore therapeutic possibilities are limited at the moment. For therapeutic approaches it is essential to gain further knowledge about the main risk factors and molecular mechanisms in the pathogenesis of T2DM and related complications.

**Figure 1:**
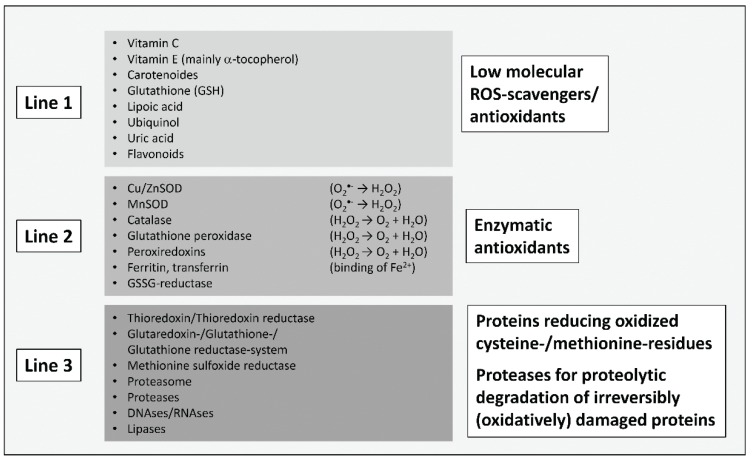
“Three lines of antioxidative defense” in mammalian cells, modified according to [5]. The first line contains low molecular antioxidants that can scavenge ROS/reactive particles in a purely competitive way, preventing damage from cellular structures like proteins, nucleobases and lipids. The reaction products are mostly significantly less reactive and may in several cases be restored by cellular systems (like vitamin C and tocopherol in a glutathione (GSH)-consuming manner). The most important and abundant low molecular intracellular scavenger is GSH, thus determining the cellular redox-state, defined as the ratio of GSH to its oxidized form, glutathione disulfide (GSSG). Under normal physiological conditions, this ratio is about 1:1000 (GSSG:GSH) or even higher, providing a strongly reducing cellular environment. The second line of defense contains antioxidative enzymes that are able to convert ROS into less reactive particles. This includes the superoxide dismutases (Cu, ZnSOD and MnSOD) as well as catalase. Further important enzymes are glutathione peroxidases, catalyzing the reaction of peroxides (R-OOH) to hydroxyls (R-OH) via GSH-consumption. Besides catalase, glutathione peroxidases are the most important H_2_O_2_-detoxifying enzymes. In this group enzymes are also found, which bind redox-active metals—iron is the most important transition metal in mammalian cells—in an inert form. Otherwise, metals like iron (Fe^2+^) or copper (Cu^+^) are able to transfer an electron to H_2_O_2_ (Fenton reaction)_,_ releasing both OH^−^ and the highly reactive hydroxyl radical (^•^OH). ^•^OH is able to oxidize virtually every organic molecule. The oxidized forms of those metals (Fe^3+^/Cu^2+^) are quickly reduced in the cytosolic environment, fuelling the vicious circle. In the last line of defense is a summary of enzymes that are able to restore oxidatively modified amino acids (only methionine and cysteine), thus preventing proteolytic degradation of the whole damaged protein. If repair is not possible, several proteases are available, that can recognize and remove dysfunctional proteins in a proteolytic manner, preventing their intracellular accumulation. The most important one is the proteasomal system, responsible for the degradation of more than 90% of all (oxidatively) damaged proteins, as well as the cathepsins of the lysosomal system. Other catabolic enzymes might also play some role in these defense lines.

The formation of reactive oxygen species (ROS) is an inevitable byproduct of metabolism. The main source of ROS in mammalian cells is the “dripping” of electrons from the mitochondrial respiratory chain, and their subsequent transfer to molecular oxygen, resulting in the formation of the superoxide anion (O_2_^•−^). Together with hydrogen peroxide (H_2_O_2_) and nitric oxide (^•^NO), superoxide is considered to be one of the main “primary” ROS, forming the bulk variety of other ROS found in cells in further reactions. Due to the highly reducing cellular environment, powerful antioxidative systems are needed, that are capable of scavenging ROS or transforming them into less reactive products. Another task of the cell’s antioxidative machinery is the “repair” of already (oxidatively) damaged structures or their degradation. Thus, “three lines of defense” can be defined roughly (as shown in Figure 1): low molecular ROS-scavengers, antioxidative enzymes, and repairing or degrading ones. If both, the antioxidative machinery of a cell is overwhelmed by the present amount of ROS and the cellular redox-signaling is disturbed, the cell is found in a stage defined as “oxidative stress” [4]. Oxidative stress, induced by an abundance of ROS or failure in the antioxidative machinery, is the cause of many pathologies.

There is increasing evidence that oxidative stress also plays a key role in pathological processes observed in T2DM (reviewed in [6,7,8]). Oxidative stress has long been associated with diabetic complications and more recent studies indicate that oxidative stress is also causal in the development of β cell dysfunction and insulin resistance, the two hallmarks of T2DM. Beta cell dysfunction and insulin resistance occur long before blood glucose levels reach the amount defined as prediabetes [9]. Moreover, both processes mediate progression of prediabetes to diabetes so that the prevention of insulin resistance as well as of β cell failure is essential to prevent T2DM. Diabetes is related to oxidative stress at least partly to the overproduction of ROS. Under diabetic conditions, there are several sources of ROS described, among them advanced glycation end products (AGEs). AGEs are a group of heterogeneous compounds increasingly formed under hyperglycemic conditions. Due to this and their damaging potential, AGEs have been assumed to be involved in the pathogenesis of T2DM and diabetic complications. Additionally, it was recently proposed that another source of AGEs, the diet, contributes to the development of T2DM (reviewed in [10]). This review will give an overview on the involvement of AGEs in T2DM, in particular in the development of insulin resistance, β cell dysfunction and death as well as their role in diabetic complications.

## 2. AGEs in Diabetes: An Overview

**Figure 2:**
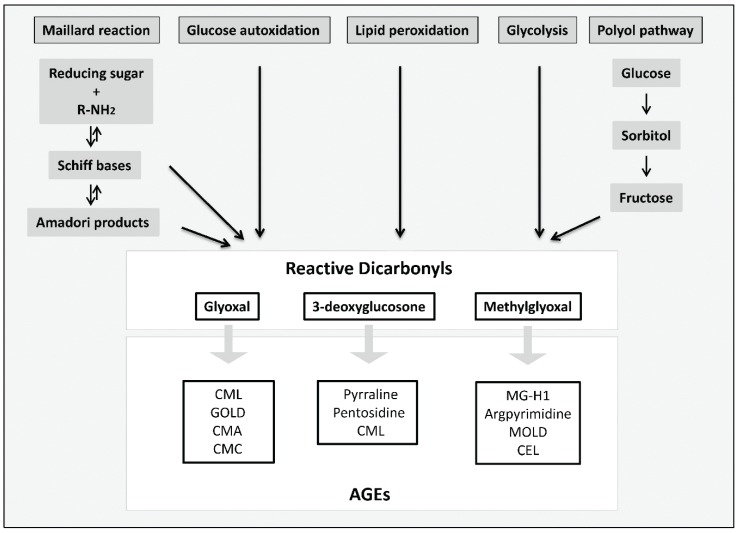
Formation of reactive dicarbonyls and AGEs, modified according to [29]. Reactive dicarbonyls including methylglyoxal, glyoxal and 3-deoxyglucosone are formed through several pathways: the Maillard reaction, the polyol pathway, glycolysis, lipid peroxidation or glucose autoxidation. Dicarbonyl compounds react further to form irreversible products, the so-called AGEs.

The first link between glycated proteins and diabetes was made in 1968 with the discovery of an altered form of hemoglobin (meanwhile known as HbA1_c_) in red blood cells of patients with diabetes [11]. It became clear, that glycation occurs predominantly on the N-terminal valine of the β chain and that this Amadori product is formed non-enzymatically in a reaction which was before only known to take place in food [12,13]. In this so-called Maillard reaction, the carbonyl group of a reducing sugar reacts with the amino group of a protein, lipid or nucleic acid generating Schiff bases which rearrange to Amadori products (Figure 2). However, Amadori products are relatively unstable so that further consecutive and parallel reactions occur, eventually leading to the formation of irreversible AGEs. The Maillard reaction is the most common pathway known to form AGEs. Not only during all stages of the Maillard reaction, but also as intermediates or byproducts of glucose autoxidation, lipid peroxidation or the polyol pathway, high reactive carbonyl compounds, including glyoxal, methylglyoxal or 3-deoxyglucosone are formed [14,15,16]. Increased concentrations of glyoxal, methylglyoxal as well as 3-deoxyglucosone have been found in plasma of patients with T2DM [17]. Glyoxal, for example, causes the formation of Nε-(carboxymethyl) lysine (CML) [18] which is at present the best characterized AGE. Further AGEs formed by glyoxal are glyoxal-derived lysyl dimer (GOLD) [19], Nω-(carboxymethyl) arginine (CMA) [20] or S-carboxymethylcysteine [21]. Methylglyoxal causes the generation of, for example, Nε-(carboxyethyl) lysine (CEL) [22], methylglyoxal-derived lysyl dimer (MOLD) [23], argpyrimidine [24] or methylglyoxal-derived hydroimidazolone MG-H1 [25] whereas 3-deoxyglucosone leads to the formation of pyrraline [26], pentosidine [27], imidazolone or also CML [28].

Even if oxidation is not always necessary, many AGEs are generated by a combination of oxidation and glycation so that the formation of so-called glycoxidation products is triggered by oxidative stress [30]. Two important AGEs produced by glycoxidation are pentosidine and CML. The complexity and diversity of AGE formation makes clear why substances belonging to the group of AGEs are so heterogeneous regarding their chemical and physical properties. Some AGEs are fluorescent; a few induce protein cross-linking. There are compounds which show both properties, other AGEs are either fluorescent or cross-linkers. AGE formation occurs intra- as well as extracellularly as part of physiological metabolism. To detect AGE formation in fluids and tissues, AGE-specific fluorescence can be measured. The majority of so far identified AGEs are characterized by fluorescence in the area around an excitation wavelength of 370 nm and an emission of 440 nm [24,31,32]. Additionally, pentosidine emits light at 385 nm when excited at 335 nm [33]. Further methods for AGE detection used in *in vitro* and *in vivo* studies are immunohistochemical staining or enzyme-linked immunosorbent assay (ELISA) using antibodies against different AGEs, for example, CML or pentosidine. However, application of these methods is often limited due to lack of reliable antibodies. More sensitive methods for AGE detection include high performance liquid chromatography (HPLC), gas chromatography or liquid chromatography with different detectors (reviewed in [34]).

In people with diabetes, AGE formation is accelerated due to increased concentration of circulating glucose, AGE precursors and oxidative stress. In serum and tissues of patients with type 1 diabetes mellitus (T1DM) as well as T2DM, increased levels of AGEs, among them CML [35,36,37], MG-derived hydroimidazolone [38], pentosidine [39] or glucosepane [40,41] have been found. Furthermore, accumulation of AGEs in diabetic tissue was shown to correlate with diabetic complications (reviewed in [42]).

At this point it should be noted, that another source of AGEs, the diet, may contribute to pathological features related to diabetes. Different studies have investigated the AGE content in food items, for example by measuring CML concentrations with ELISA [43] or further ultra pressure liquid chromatography with mass spectrometry detection (UPLC-MS) [44]. That the detection of AGEs with only one method is not sufficient and can result in under- or overestimation of AGEs in food items [34], is demonstrated by comparing these studies. Goldberg *et al.* reported that food items rich in fat and protein contain particularly high concentrations of CML in contrast to carbohydrate-rich food in which only low CML concentrations were measured. In contrast, CML detection with UPLC-MS observed highest levels of CML in bread crust and evaporated full-fat milk while lowest CML levels were detected in uncooked minced beef, raw full-fat milk and pasteurized skimmed milk. No CML was detected in olive oil. Nevertheless, people are continuously exposed to AGEs through their diet, and this source might even be greater than the amount formed endogenously [45]. Around one-tenth [46] or even more [47] of the consumed AGEs are absorbed in the gut and, therefore, contribute to the body’s AGE pool. Studies reported that the dietary uptake of AGEs correlates with serum AGE levels [46,48] and that restriction of food-derived AGEs can lower AGE concentrations in serum [49,50]. There is a growing body of evidence that the amount of food-derived AGEs which contributes to the AGE pool is sufficient to promote the development of T2DM. Studies in humans and mice revealed that an AGE-rich diet elevates AGE concentrations such as MG-H1 also in non-diabetic subjects and increases biomarkers of inflammation and oxidative stress associated with insulin resistance [51,52]. Furthermore, it was shown that a high-AGE diet causes decreased insulin secretion and increased β cell death in rats [53]. Taken together, AGEs are proposed to play a role in the development and progression of T2DM as well as in diabetic complications.

The damaging potential of AGEs results from direct alterations on protein structures and functions due to AGEs *per se* or the cross-linking effect of some AGEs. AGEs are often found in the extracellular matrix (ECM) and thus modified matrix proteins impair matrix-matrix as well as matrix-cell interactions. This may cause cell death, cell differentiation or reduced cell adhesion and migration. Intracellular proteins are also targets of modifications and AGE formation was shown to impair their functions. Besides direct changes in protein structures and functions, AGE-mediated damage occurs via binding of AGEs to the receptor of advanced glycation end products (RAGE). RAGE belongs to the immunoglobulin superfamily and additionally interacts with a wide range of ligands including S100 calgranulins [54], high mobility group box 1 [55], or Mac-1 [56] which is why RAGE is also classified as a multi-ligand receptor. In recent years the interaction of AGEs with RAGE was studied *in vitro* demonstrating the activation of Janus kinase, rho-GTPases, extracellular-signal-regulated kinase 1/2 and p38 mitogen-activated protein kinase due to AGE-RAGE interaction [57,58,59,60]. It should be pointed out that ligand binding to RAGE activates NAPDH oxidases and thus increases intracellular ROS formation [61,62]. Increased ROS in turn leads to AGE formation, which triggers all described damaging mechanisms mediated by AGEs, but also activates the transcription factor nuclear factor kappa B (NFκB) [63]. Activation of NFκB increases the expression of proinflammatory cytokines such as interleukin 6 [64] and monocyte chemoattractant peptide 1 (MCP-1) [65] as well as RAGE itself [66] thus intensifying the inflammatory response. There are two possibilities to protect tissues against AGE-mediated damage: AGEs are eliminated or cells activate compensatory mechanisms. It has been shown that cells process other AGE receptors which are able to bind extracellular AGEs and mediate their cellular uptake (reviewed in [67]). In cells, AGEs can be degraded by the endosomal-lysosomal system. Cathepsin D and L were identified as two enzymes involved in the detoxification of AGEs [68,69]. After digestion, the AGE degradation products are released and circulate in the bloodstream until renal elimination. One possibility to prevent RAGE-mediated damage, is enabled by the upregulation of advanced glycation end products receptor 1 (AGER1) after AGE exposure. Although AGER1 was first only associated with the AGE turnover, studies observed that its upregulation suppresses RAGE-mediated pathways: AGER1 inhibits the activity of NADPH oxidase and weakens oxidative stress generation as well as ROS-mediated signaling [70,71,72,73]. Moreover, it has been described that AGER1 is linked to sirtuin1 (SIRT1) [50,52], a NAD^+^-dependent deacetylase. For example, through deacetylation of NFκB SIRT1 suppresses NFκB-mediated proinflammatory processes. However, long term exposure to AGEs depletes AGER1 and SIRT1 expression [52] so that the regulation of RAGE signaling fails and oxidative stress and inflammation increase.

## 3. Advanced Glycation End Products and Insulin Resistance

Insulin resistance describes the condition when cells are no longer able to appropriately respond to the hormone insulin, which mediates the uptake of glucose. Although not all persons with insulin resistance develop T2DM, it is one relevant factor which increases the risk to develop diabetes [74]. In turn, genetic as well as environmental factors, especially obesity and lack of physical activity, increase the risk that cells become insulin resistant. There is more and more evidence that AGEs are another risk factor for the development of insulin resistance. For example, by multiple regression analysis Tan *et al.* reported that AGE levels are independently correlated with insulin resistance in healthy subjects [75]. Insulin resistance was estimated by the homeostatic model assessment index (HOMA-IR) which is based on the calculation of fasting insulin and glucose concentration [76]. Another study by Tahara *et al.* in which more than 300 non-diabetic persons were examined confirmed that serum AGE levels were independently correlated with the HOMA-IR [77].

**Figure 3:**
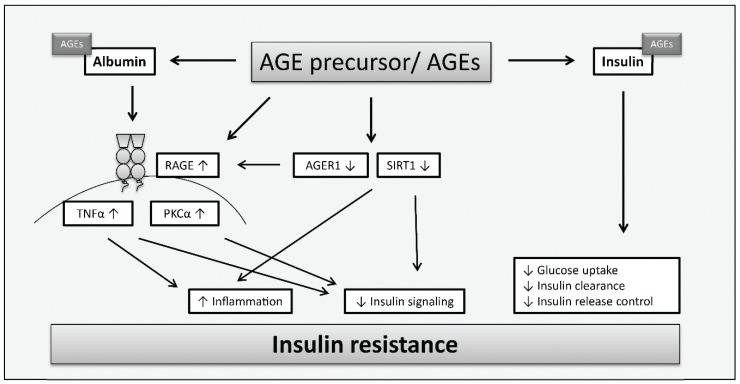
Mechanisms of AGEs leading to insulin resistance in insulin-sensitive tissues according to [52,78,79,80,84,85,86]. AGEs are involved in mechanisms contributing to insulin resistance due to direct modification of insulin which alters insulin action resulting in impaired glucose uptake, inhibited insulin clearance or further increased insulin secretion. Furthermore, AGEs may contribute to insulin resistance via increased expression of RAGE and reduced expression of AGER1 and SIRT1. AGEs affect insulin signaling and trigger inflammation via stimulation of PKCα and upregulation of TNFα. SIRT1 depletion causes changes in insulin signaling and induces inflammation.

The underlying molecular mechanisms leading to AGE-induced insulin resistance are still poorly understood, but some *in vitro* and *in vivo* studies were performed to gain knowledge in this research area. Figure 3 summarizes the principle mechanisms of AGE contribution to insulin resistance which have been observed in the described studies. As serum proteins are frequent targets of modifications by sugars and reactive carbonyl compounds such as methylglyoxal and glyoxal, *in vitro* glycated albumin is often used for cell culture experiments. Glycated albumin was shown to induce the expression of tumor necrosis factor alpha (TNFα) which suppresses insulin signaling [78]. Furthermore, protein kinase C alpha (PKCα) was reported as a target of glycated human serum albumin leading to increased serine/threonine phosphorylation of insulin receptor substrate (IRS) 1 and 2 but reduced IRS tyrosine phosphorylation. This resulted in impaired insulin signaling (phosphatidylinositol 3-kinase/protein kinase B pathway) and inhibition of insulin-mediated glucose metabolism [79]. As the induction of PKCα via protein tyrosine kinase Src and the inhibition of insulin receptor substrate 1 (IRS1) was mediated by RAGE after the incubation of skeletal muscle cells with glycated albumin, it is assumed that RAGE plays a role in this process [80]. Previous studies already showed that both, TNFα (reviewed in [81]) and proteins of the PKC family [82,83], are involved in insulin resistance.

Another possible factor contributing to insulin resistance is the direct glycation of insulin. Although insulin has a very short half-life and is not a typical target for modification, glycation sites on insulin have been found *in vivo* [85,87] as well as when cells and islets were cultured under hyperglycemic conditions [87,88]. *In vitro*, glycation of human insulin was shown to occur in the phenylalanine position in the amino terminus of the insulin β chain [89]. Moreover, it was demonstrated that the reaction of glyoxal with insulin results in the formation of N-terminal pyrazinones, products belonging to AGEs [90]. Studies using *in vitro* prepared monoglycated insulin revealed that glycation affects insulin function. When *in vitro* monoglycated insulin was infused to mice the glucose lowering potential was around 20% lower compared to unmodified insulin [84]. Cell culture experiments with isolated muscle and glycated insulin additionally showed that glucose uptake, oxidation and glycogen production is reduced. Monoglycated insulin had similar effects in humans. After infusion to healthy humans, around 70% more glycated insulin was needed to induce an equal amount of glucose uptake compared to unmodified insulin [85]. The role of glycated insulin in T2DM was supported by the measurement of the modified insulin in the plasma of T2DM subjects which was found to comprise around 9% of the total insulin level [85]. Furthermore, it was shown that methylglyoxal causes modifications on the arginine residue of the insulin β chain decreasing glucose uptake in insulin-sensitive cells including 3T3-L1 adipocytes and L8 skeletal muscle cells [86]. Usually, extracellular insulin concentrations regulate insulin secretion from β cells, however, methylglyoxal-modified insulin can no longer inhibit insulin secretion. Moreover, Jia *et al.* reported that methylglyoxal-modified insulin triggers hyperinsulinemia through decreased insulin clearance by liver cells [86].

The potential role of AGEs in insulin resistance is supported by studies examining the influence of antiglycating agents on the manifestation of insulin resistance. The agents pyridoxamine or TM2002 inhibit the formation of AGEs and were shown to improve insulin resistance in rodents which already suffered from diabetes [91] or in which insulin resistance was induced by the administration of methylglyoxal [92]. Antioxidants reduced insulin resistance in the same manner making it difficult to distinguish between oxidative stress and AGE formation but making clear that there is an important link between both factors.

There are studies which indicate that predominantly prooxidative AGEs derived from the diet increase the risk for insulin resistance. Cai *et al.* reported that a diet enriched with methylglyoxal-modified albumin (MG-diet) and fed to non-obese C57BL6 mice led to weight gain, adiposity and the development of insulin resistance in F3/MG^+^ mice [52]. The MG-diet increased serum AGE levels and AGE-lipids. Furthermore, the chronic AGE intake caused elevated oxidative stress and inflammation and resulted in an insulin-resistant state (increased level of fasting plasma insulin and leptin and reduced adiponectin level). The phenotypical change was mediated by reduced expression of AGER1 and SIRT1 and upregulation of RAGE in skeletal muscle, liver and white adipose tissue as well as an increase in NFκB acetylated p65 in adipocytes. This resulted in changes in insulin signaling and increased inflammation leading to elevated levels of oxidative stress. In this context, another study showed that restriction of AGEs in a cohort of patients with T2DM reduced insulin resistance and increased expression of SIRT1 and AGER1 [50]. Due to its deacetylase activity, SIRT1 seems to have a significant role in insulin actions. On the one hand, SIRT1 is involved in the induction of insulin secretion from β cells, on the other hand SIRT1 induces insulin signaling by inhibiting negative regulators or further regulating the activation of IRS1 and 2 and Akt. Moreover, as SIRT1 also regulates inflammatory processes, adiponectin secretion or ROS formation, it indirectly contributes to the development of insulin resistance [93].

## 4. The Role of AGEs in β Cell Dysfunction and β Cell Death

There is a growing body of evidence that AGEs not only contribute to insulin resistance, but also damage β cells directly, leading to impaired functions or even cell death. The cytotoxic potential of AGEs on pancreatic β cells was investigated in a number of studies. Lim *et al.* reported that AGE treatment caused apoptosis in β cells [94]. Additionally, they showed that AGEs stimulate ROS production and induce the expression of RAGE. Inhibition of RAGE as well as antioxidant treatment prevented these changes so that AGEs might induce apoptosis via RAGE-induced ROS formation. However, they also demonstrated that proliferation increases after AGE treatment. In contrast, decreased proliferation but also ROS-induced cell death in HIT-T15 cells due to treatment with ribose-modified serum was observed by Viviani *et al.* [95]. Moreover, Zhu *et al.* indicated that apoptosis in β cells characterized by caspase activation, cytochrome c release and reduced expression of anti-apoptotic bcl2 might be due to RAGE [96]. In the study of Lin *et al.*, INS-1 cells were treated with AGEs resulting in cell apoptosis [97]. They concluded that the AGE-induced ROS generation occurs primarily through the mitochondrial electron transport chain but also through stress-related signaling pathways (Jun N-terminal kinase and p38) which activates ROS production via the NAPDH oxidase.

**Figure 4:**
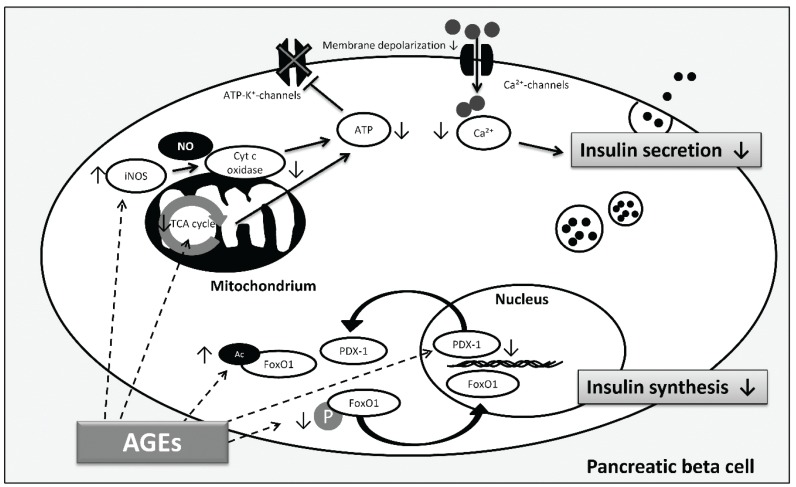
AGE-induced pathways involved in β cell dysfunction according to [98,100,102,103]. Decreased insulin synthesis and reduced insulin secretion are both involved in β cell failure contributing to hyperglycemia. AGEs reduce phosphorylation (P) and induce acetylation (Ac) of FoxO1, thus, FoxO1 translocates into the nucleus and is protected against proteasomal degradation, respectively. In addition, AGEs induce PDX-1 translocation into the cytoplasm and decrease PDX-1 protein expression, finally affecting insulin gene transcription and insulin synthesis. Regarding insulin secretion, AGEs cause inhibition by activation of iNOS and consequent blocking of cytochrome c oxidase activity and ATP depletion. Moreover, AGEs decrease insulin secretion through alterations in the TCA cycle which limits ATP production. ATP depletion inhibits closure of ATP-dependent potassium channels which leads to reduced membrane depolarization and decrease of intracellular calcium concentration inhibiting insulin secretion. (Arrows illustrate direct interactions; dashed arrows illustrate possible targets of AGEs).

In addition to β cell death, most of the studies showed that AGEs affect insulin secretion [95,96,97]. Further evidence for the AGE-mediated decline in insulin secretion was given by Zhao *et al.* [98]. They showed that AGEs block the activity of cytochrome c oxidase and production of adenosine triphosphate (ATP) in islets isolated from mice. Impaired insulin secretion increases plasma glucose levels which were accompanied by increased formation of **^•^**NO and elevated expression of inducible nitric oxide synthase (iNOS) suggesting that AGEs cause the induction of iNOS so that increasing concentrations of **^•^**NO inhibit cytochrome c oxidase activity and ATP production (Figure 4). ATP is necessary for insulin secretion as ATP causes the shutdown of ATP-sensitive potassium channels leading to membrane depolarization and the influx of Ca^2+^. Increased intracellular Ca^2+^-concentrations trigger the exocytosis of insulin granules [99]. Low ATP levels inhibit this process. More recently, Hachiya *et al.* tested the influence of bovine serum albumin (BSA) modified with glucose and glyceraldehyde on insulin secretion of isolated rat pancreatic islets [100]. Both, glucose-BSA and glyceraldehyde-BSA impaired insulin secretion induced by high concentrations of glucose. However, the authors were not able to detect any changes in the expression of oxidative response genes including iNOS. They concluded the impaired insulin secretion to be due to defects in the tricarboxylic acid cycle (TCA). Moreover, gene expression of the NADH (reduced nicotinamide adenine dinucleotide) shuttle enzymes malate dehydrogenase 1/2 was reduced after AGE treatment. However, as the expression of glycerol phosphate shuttle was unchanged, and inhibition of both have been linked to decreased insulin secretion [101], further investigations must clarify whether AGEs affect NADH shuttle function and thus ATP production. The different results reveal that more research to understand the causal mechanisms leading to impaired insulin secretion needs to be performed.

Another factor contributing to reduced insulin secretion is the decline in insulin gene transcription. In this respect, AGEs are also proposed to play a role (Figure 4). Shu *et al.* reported the impaired insulin secretion of β cells as a result of the downregulation of insulin transcription [102]. They identified that the transcription factor FoxO1 (Forkhead box protein O1) accumulates in the nucleus which in turn decreases the expression of the transcription factor PDX-1 (pancreatic and duodenal homeobox-1) by reducing protein stability. This is in agreement with the study of Puddu *et al.* who observed that the reduced insulin content in the pancreatic islet cell line HIT-T15 after AGE treatment is linked to reduced expression of PDX-1 and an increase of FoxO1 in the nucleus [103]. They suggested that AGEs decrease phosphorylation of FoxO1, thus, inducing the translocation into the nucleus. In addition, they were able to show that AGEs induce FoxO1 acetylation and that PDX-1 translocates into the cytoplasm. Acetylation of the transcription factor protects against proteasomal degradation, and nucleocytoplasmic translocation of PDX-1 decreases its availability for insulin transcription. For therapeutic approaches, it is important to note that even if hyperglycemia is restored, AGEs can still contribute to β cell failure and thus T2DM. Therefore, destroying AGE structures, preventing endogenous AGE formation as well as dietary AGE uptake may all be considered as part of a therapy to completely “remove” sources of β cell dysfunction.

## 5. The Role of AGEs in Diabetic Complications

Diabetics have an increased risk for the development of infections and several diseases including cardiovascular, kidney, eye, nerves and skin diseases. Referring to the International Diabetes Federation, diabetes is the main cause of blindness, amputation of the lower limb, kidney failure and cardiovascular diseases in many countries [2]. Most relevant for the development of diabetic complications is the exposure to hyperglycemia. One of the most important injuries which arise from hyperglycemia is damage to the vascular system. If injury occurs on large or small blood vessel most diabetic diseases can be accordingly grouped into macro- or microvascular complications, respectively. Several mechanisms leading from hyperglycemia to diabetic diseases have been described, among them the formation of AGEs.

AGE formation and accumulation increasingly occurs under diabetic conditions, and even if glycemic control is restored, AGEs can remain in tissues of diabetic subjects for a long time. Diabetic complications occur in both types of diabetes, T1DM and T2DM, and in general, there is evidence that AGEs accumulating in tissues and serum are associated with diabetic complications [42]. Regarding T2DM, studies were able to show that AGE-levels correlate with diabetic complications including diabetic retinopathy [104,105,106], nephropathy [107,108] and cardiovascular disease [35,107,109]. Most of the studies used glycated serum proteins to investigate their relevance as a marker or even a predictor for diabetic diseases. Frequently, they measured AGE-levels by immunoassay (ELISA, DELFIA) using anti-AGE, anti-CML or anti-hydroimidazolone antibodies. These methods are not very specific and the measurements of plasma AGEs may lead to underestimated associations between AGEs and diabetic complications due to the fact that most AGEs are formed or further accumulate intracellular or in tissues without reaching the circulation. This might explain why, for example, the study of Busch *et al.* reported no association of CML with cardiovascular and renal outcomes in T2DM patients [110]. However, even by analyzing CML, CEL and pentosidine with liquid chromatography, a more precise technique to measure AGEs, no association between AGEs and prior cardiovascular events was observed in a cross-sectional study [111]. Indeed, more recently the same group published results of a prospective study in which higher plasma levels of CML, CEL and pentosidine were associated with an increased risk of cardiovascular outcome in T2DM patients [112]. Although the causal relationship needs to be further clarified there is increasing evidence observed in these clinical studies that AGEs serve as potential biomarkers for diabetic complications.

### 5.1. Modified ECM Proteins and the Relevance for Diabetic Complications

**Figure 5:**
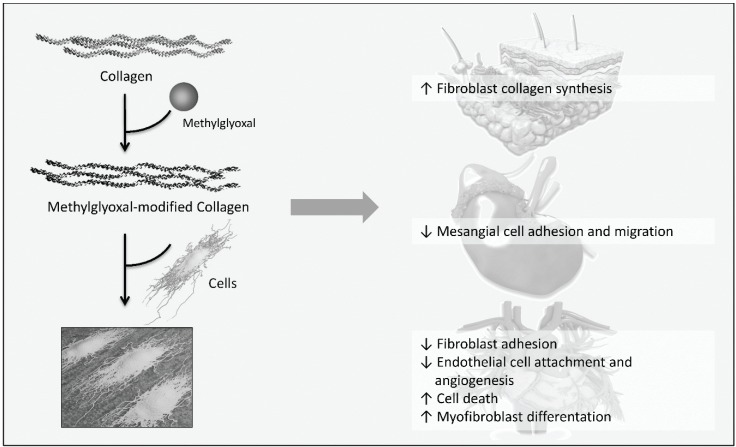
Influence of methylglyoxal-modified collagen on cellular functions relevant to diabetic complications, according to [113,116,119,120]. Cells were cultured on methylglyoxal-modified collagen and the effect of AGE-collagen on cellular functions was studied. Dermal fibroblasts grown on modified collagen increase collagen synthesis. Methylglyoxal-modified collagen reduces cell adhesion and migration of mesangial cells, cardiac fibroblast are less adherent. Myofibroblast differentiation is stimulated in cardiac fibroblasts cultured on modified collagen and modifications of basement membrane collagen causes detachment, cell death and reduced angiogenesis of vascular endothelial cells.

One main target of increased concentrations of reducing sugars and dicarbonyl compounds found in diabetes is collagen. There are a few studies which investigated the effect of modified collagen on cell functions. According to the subsequent studies, a simplified scheme describing which cell functions are affected by methylglyoxal-modified collagen, contributing to diabetes-related changes related to diabetic complications, is illustrated in Figure 5. Firstly, it was reported that modification of basement membrane collagen type IV by methylglyoxal reduces attachment of vascular endothelial cells and angiogenesis [113]. AGEs such as MG-H1 were found on integrin-binding sites of collagen and were assumed to cause decreased cell attachment. As demonstrated by Chong *et al.*, methylglyoxal modifications of arginine residues in the integrin-binding sequence also reduce collagen binding so that collagen degradation by phagocytosis is impaired, thus, promoting fibrosis [114]. Regarding the collagen turnover, it was shown that collagen modified with glyoxal and methylglyoxal is *per se* less degradable by proteases due to the formation of cross-links [115]. Yuen *et al.* observed that methylglyoxal-modified collagen type I inhibits cell adhesion of cardiac fibroblasts while stimulating myofibroblast differentiation and their migration [116]. Further investigations of Talior-Volodarsky *et al.* reported that myofibroblast differentiation is stimulated by upregulation of α11 integrin expression which was found to be induced by transforming growth factor (TGF) β2/Smad3 signaling [117,118].

Myofibroblasts mediate fibrosis, a pathological response to tissue damage which has been associated with diabetic complication and heart failure due to fibrotic lesions. That modified collagen alters cell-matrix interaction was also shown by Pozzi *et al.* [119]. On the one hand, cell adhesion and migration but not proliferation of mesangial cells plated on collagen type IV modified with methylglyoxal is inhibited. On the other hand, long term glucose-modified collagen reduces migration and proliferation and increases collagen synthesis. The authors proposed that these findings may explain pathological mechanisms seen in diabetic nephropathy: early proliferation of mesangial cells followed by impaired proliferation, mesangial matrix expansion and mesangial cell dysfunction. In the study of Sassi-Gaha *et al.* [120], after modification of type I collagen lattices with methylglyoxal and 3-deoxyglucosone, dermal fibroblast were seeded on the modified collagen. For methylglyoxal an increased expression of TGFβ1, collagen and β1 integrin, and decreased expression of the transcription factor Smad7 was shown. The opposite effects were found for fibroblasts grown on 3-deoxyglucosone-modified collagen. To understand the pathological mechanisms mediated by AGEs it is quite important to investigate the effect of different AGE precursors as well as individual AGEs.

In addition to collagen, AGE formation on further ECM protein including laminin or fibronectin has been studied showing impaired self-assembly and interactions with other matrix components as well as matrix-cell interactions [121,122,123,124,125]. It is assumed that glycation of these ECM proteins, therefore, may contribute to diabetic complications. Just to give an example, bovine retinal pericytes cultured on fibronectin modified with glyoxal and methylglyoxal were shown to induce apoptosis which may contribute to cell loss in diabetic retinopathy [124]. Regarding the development of diabetic neuropathy, glycated laminin and fibronectin reduced neurite outgrowth providing a mechanism for the failure of axonal regeneration and collateral sprouting [125].

### 5.2. Ischemic Heart Disease and Atherosclerosis

As already mentioned, patients with diabetes are at high risk of developing cardiovascular disease [126]. One common type of cardiovascular diseases is ischemic heart disease in which the blood supply to the heart is reduced often due to plaque formation in the arterial wall. The association between AGEs and ischemic heart disease of patients with T2DM was investigated in some clinical studies. Kilhovd *et al.* reported increased serum AGEs in diabetic persons with ischemic heart disease compared to patients without [35]. This is in agreement with a study in which increased serum AGE concentrations were measured in patients with coronary artery disease compared to diabetic patients without coronary artery disease [109]. Furthermore, serum AGE levels may predict ischemic heart disease mortality in persons with T2DM. Evidence was given in a long-term follow-up study in which serum AGEs, measured by immunoassay using a polyclonal anti-AGE antibody, were predictors for total, cardiovascular as well as coronary mortality in woman with T2DM [127]. Moreover, skin autofluorescence has been strongly related to coronary heart disease and mortality in patients with T2DM [128]. The measurement of skin autofluorescence is a technique with some limitations for AGE analysis: not all AGEs are fluorescent, no specific compound is measured and there is no information on the quantity of AGEs at all. However, skin autofluorescence can be used as a non-invasive method and is associated with AGE accumulation *in vivo* [129].

Atherosclerosis is the underlying cause of most ischemic heart diseases and it has been suggested that AGEs are involved in the development of atherosclerosis (for more detailed review see [130]). On the one hand it has been shown that AGEs accumulate in atherosclerotic lesions [131,132] and on the other hand studies identified AGE-mediated mechanisms related to endothelial dysfunction, inflammation and lipid modifications. In particular, it was shown that AGEs contribute to endothelial dysfunction through their pro-apoptotic effect on endothelial cells [133,134] and endothelial progenitor cells [135]. Moreover, AGEs stimulate the expression of genes such as MCP-1, intercellular adhesion molecule 1, vascular cell adhesion molecule 1 and plasminogen activator inhibitor 1 [136,137,138,139,140,141]. This mediates recruitment and adhesion of inflammatory cells to the vessel wall or further inhibition of fibrinolysis. The expression of MCP-1 and adhesion molecules can be induced by endothelin 1, its expression in turn is also stimulated by AGEs [142]. In porcine coronary fibroblasts, it was observed that AGEs induce the mRNA expression of interleukin 6, vascular cell adhesion molecule 1 and MCP-1 followed by increased interleukin secretion and leukocyte adhesion [143]. The atherogenic properties of AGEs could be further attributed to the ^•^NO quenching effect of AGEs [144]. Furthermore, AGEs impair the synthesis of ^•^NO by decreasing the expression as well as the activity of endothelial ^•^NO synthase [145,146]. ^•^NO mediates a series of intracellular effects that lead to endothelial regeneration, vasodilation and inhibition of platelet adhesion and leukocyte chemotaxis and therefore defects in ^•^NO production and activity are supposed to be major mechanisms of endothelial dysfunction and atherosclerosis [147].

To further clarify the role of AGEs in diabetic complications, *in vitro* and *in vivo* studies showing AGE-mediated pathomechanisms which contribute to diabetic retino- and nephropathy will be highlighted below.

### 5.3. Diabetic Retinopathy

AGE-albumin-induced pericyte death has been assumed to be caused by AGE-RAGE interaction [148]. Moreover, studies reported that AGEs induce ROS formation and reduce protein kinase B/Akt [124] or further platelet-derived growth factor signaling [149] contributing to reduced pericyte survival. Pericytes are cells which cover capillaries in the retina and their loss is an early event in diabetic retinopathy followed by the formation of acellular capillaries (capillaries devoid of cells), microaneurysms and vascular basement membrane thickening. The interaction of pericyctes with endothelial cells is necessary for the maintenance of the blood-retina barrier [150] so that pericycte loss is also associated with the breakdown of the barrier.

The blood-retina barrier regulates the flux of nutrients, fluids and other blood components into the retina and its breakdown may cause the development of macular edema which is a main cause for vision loss in diabetes. Another mechanism leading to the breakdown of the blood-retinal barrier is the induction of retinal leukostasis, a state of chronic inflammation, which may contribute to endothelial cell death and increased vascular permeability. The involvement of AGEs in this process has been shown by Moore *et al.* [151]. AGEs induce NFκB DNA binding, intercellular adhesion molecule 1 expression and further leukocyte adhesion to retinal endothelial cells. *In vivo*, AGE-albumin injection into mice resulted in a breakdown of the blood-retina barrier [151]. Moreover, the breakdown of the blood-retina barrier has been shown to be dependent on VEGF. AGE-upregulation of vascular endothelial growth factor (VEGF) expression was observed after AGE-albumin injection into rodents, as well as after AGE-albumin incubation of cultured retinal cells [148,152,153,154], leading to vascular permeability and angiogenesis.

### 5.4. Diabetic Nephropathy

Similar to pericytes in the retina, AGEs induce apoptosis and VEGF expression in mesangial cells [155], specialized pericytes which are located in blood vessels of the kidney. Mesangial cells in the glomerulus regulate glomerular filtration and provide structural support [156]. Their loss or further VEGF upregulation contributes to increased vascular permeability associated with hyperfiltration and proteinuria in kidney diseases, suggesting that AGEs play a role here. Furthermore, it has been shown, that AGEs upregulate MCP-1 expression in mesangial cells [155]. MCP-1 is a chemokine which regulates macrophage/monocyte migration and infiltration, therefore, MCP-1 upregulation by AGEs may trigger inflammation in renal tissue. Moreover, AGE-induced expression of TGFβ was demonstrated *in vitro* [157,158] and as TGFβ1 upregulation was associated with enhanced expression of ECM proteins this might be an explanation for glomerular hypertrophy *in vivo* [159]. In this context, studies reported that TGFβ expression is induced via AGE-RAGE interaction [160,161]. Elevated expression of TGFβ is also known to induce epithelial-to-mesenchymal transition and AGEs were shown to stimulate transdifferentiation in a rat kidney epithelial cell line [161]. Another protein which plays a role in AGE-induced epithelial-to-mesenchymal transition is prosclerotic connective tissue growth factor (CTGF), a target gene of TGFβ signaling which is elevated in plasma of persons with diabetic nephropathy [162,163]. Because AGEs induce epithelial-to-mesenchymal transition and CTGF is a downstream target of TGFβ it is generally believed that increased CTGF expression is mediated through TGFβ/Smad signaling. More recently, AGE stimulation of tubular CTGF expression was also found to be independent of TGFβ/Smad signaling. This pathway is RAGE dependent and leads to the activation of Smad3 through the induction of extracellular-signal-regulated kinase 1/2 and p38 [164].

## 6. Conclusions

Extensive research has been performed in the last years to clarify the impact of AGEs as well as to identify causal pathological mechanisms in the development and progression of T2DM (insulin resistance, β cell death and dysfunction) and in diabetic diseases. Increasing concentrations of different AGEs in serum and tissues of diabetic persons have been detected and the association of AGEs with diabetic complications has been shown in several studies suggesting AGEs as biomarkers and even predictors for diabetic complications. Moreover, studies reported a wide range of effects and reactions induced by AGEs which were proposed to play a role in insulin resistance, β cell failure and diabetic complications. In general, it was found that AGE formation occurs on extra- and intracellular proteins leading to protein cross-linking, structural and functional changes, e.g., loss of enzyme activity. Modifications on ECM proteins have been shown to affect not only matrix-matrix but especially matrix-cell interactions. Many studies proposed that the damaging effect of AGEs is predominantly induced by RAGE signaling thus increasing ROS formation and inflammatory processes. However, there are also some critical aspects which must be considered when obtaining general conclusions from the data. Many studies used AGEs prepared *in vitro* by incubating a protein, often albumin, with a reducing sugar or dicarbonyl compound. According to Henle, only a small amount of AGEs is formed in glucose-modified BSA in contrast to Amadori products such as fructoselysine which accounts for about 90% of the detectable lysine derivatization [45]. Therefore, it is questionable whether the induced biological effects are stimulated by AGEs, Amadori products or even other modification products. Improvement of analytical methods may contribute to more precise results regarding the involvement of AGEs in general but also individual AGE structures. Because it is estimated that the wide range of AGEs is at present only partially identified, this further impedes the research about the biological effects of AGEs. Moreover, the use of different AGE precursors, different concentration and varying target proteins hinders not only comparability of studies but also complicates the understanding of AGE-induced mechanisms. Most frequently, the products are not characterized, the AGE content was measured by unspecific methods, and the individual AGEs were not identified. Without knowing the specifity of the products it is difficult to assess the findings observed in these studies and to determine the role of AGEs in several aspects of diabetes.

Another point which should be noted is that the relevance of AGE-RAGE interactions is still controversially discussed [165,166,167]. Only aggregates of highly modified AGE-proteins which are rare in tissue and body fluids are able to activate RAGE. However, these highly modified proteins can be formed exogenously in thermally processed food, thus the relevance for AGE-RAGE interaction needs to be further clarified. Nevertheless, the reduction of hyperglycemia-induced AGE formation as well as the restriction of AGE-rich food items both present targets for therapeutic approaches against the pathological events in diabetes and associated diseases. Finally, the use of AGEs as biomarkers/predictors for diabetic complications may help to reduce health problems in persons with diabetes.

## Abbreviations

AGEs: Advanced glycation end products
AGER1: Advanced glycation end product receptor 1
ATP: Adenosine triphosphate
BSA: Bovine serum albumin
CEL: Nε-(carboxyethyl) lysine
CMA: Nω-(carboxymethyl) arginine
CML: Nε-(carboxymethyl) lysine
CTGF: Connective tissue growth factor
ELISA: Enzyme-linked immunosorbent assay
ECM: Extracellular matrix
FoxO1: Forkhead box protein O1
GOLD: Glyoxal-derived lysyl dimer
GSH: Glutathione
GSSG: Glutathione disulfide
H_2_O_2_: Hydrogen peroxide
HPLC: High performance liquid chromatography
iNOS: Inducible nitric oxide synthase
IRS: Insulin receptor substrate
MCP-1: Monocyte chemoattractant peptide 1
MOLD: Methylglyoxal-derived lysyl dimer
NAD^+^: Oxidized nicotinamide adenine dinucleotide
NADH: Reduced nicotinamide adenine dinucleotide
NADPH: Reduced nicotinamide adenine dinucleotide phosphate
NFκB: Nuclear factor kappa B
**^•^**NO: Nitric oxide
O_2_**^•^**^−^: Superoxide anion
PDX-1: Pancreatic and duodenal homeobox-1
PKC α: Protein kinase c alpha
RAGE: Receptor of advanced glycation end products
ROS: Reactive oxygen species
SIRT1: Sirtuin1
T1DM: Type 1 diabetes mellitus
T2DM: Type 2 diabetes mellitus
TCA: Tricarboxylic acid cycle
TNFα: Tumor necrosis factor alpha
UPLC-MS: Ultra pressure liquid chromatography with mass spectrometry detection
VEGF: Vascular endothelial growth factor
